# When Palpitations Unmask Crista Terminalis Hypertrophy: A Case Report and Review of Current Literature

**DOI:** 10.3390/diagnostics16111615

**Published:** 2026-05-25

**Authors:** Antonia Racz, Alexandra Dădârlat-Pop, Adela Șerban, Raluca Tomoaia, Alexandru Oprea, Horia Rosianu

**Affiliations:** 1 Faculty of Medicine, Iuliu Haţieganu University of Medicine and Pharmacy, 8 Victor Babes Street, 400012 Cluj-Napoca, Romania; antoniaracz99@gmail.com (A.R.);; 2 Cardiology Department, Heart Institute Niculae Stăncioiu, 19-21 Motilor Street, 400001 Cluj-Napoca, Romania; 3 Cardiology Department, Rehabilitation Hospital, 400066 Cluj-Napoca, Romania; 4 Cardiovascular Surgery Department, Heart Institute Niculae Stăncioiu, 19-21 Motilor Street, 400001 Cluj-Napoca, Romania

**Keywords:** crista terminalis hypertrophy, right atrial mass, atrial tachyarrhythmia, diagnostic imaging, differential diagnosis, anatomical variant

## Abstract

**Background and Clinical Significance:** The crista terminalis (CT) is a physiological fibromuscular ridge in the right atrium. While benign, rare cases of CT hypertrophy present a diagnostic challenge, as it can mimic a pathological right atrial mass on cardiac imaging. The CT also presents arrhythmogenic potential and is known to be associated with right atrial tachyarrhythmias. **Case Presentation:** We present the case of a 58-year-old female that presented with rapid, irregular palpitations, accompanied by hypertension. Holter electrocardiography (ECG) confirmed self-limiting episodes of atrial tachycardia (max heart rate 170 bpm). Initial transthoracic echocardiography (TTE) identified an echogenic, non-mobile mass on the posterolateral right atrial wall. Transesophageal echocardiography (TEE) confirmed a 12 × 9 mm homogenous structure with a broad base of implantation and no intrinsic mobility, initially raising the suspicion of an atrial lipoma. Subsequent cardiac computed tomography angiography (CCTA) provided high-resolution tissue characterization, identifying the mass as a hypertrophied CT due to its precise anatomical orientation and its lack of contrast enhancement, also ruling out neoplastic and thrombotic aetiologies. **Conclusions:** CT hypertrophy is a key differential diagnosis for right atrial masses, particularly in females in their sixth decade. A multimodal imaging approach, transitioning from TTE to TEE and finally CCTA or Cardiac Magnetic Resonance Imaging (CMR), is advantageous in preventing unnecessary invasive interventions or anticoagulation.

## 1. Introduction

The crista terminalis (CT) is a C-shaped fibromuscular ridge located in the right atrium, arising during embryogenesis and forming the boundary between the primitive right atrium and the sinus venosus [1]. Clinically, the CT is important due to its ability to mimic a right atrial mass and to its arrhythmogenic potential.

In most patients, this structure regresses to an approximate size of 3–6 mm by adulthood [2]. In rare cases, the CT is hypertrophied and leads to diagnostic confusion with life-threatening conditions such as thrombi or myxomas [3]. Proper identification of this benign structure is therefore essential in avoiding unnecessary anticoagulation, invasive biopsies, or cardiac surgery.

Due to its unique electrophysiological properties, the CT is a major site of origin for right atrial tachyarrhythmias, accounting for two-thirds of cases in patients without structural cardiac abnormalities [2,4]. Mechanisms responsible for these arrhythmias include focal automaticity and conduction anisotropy, both of which are thought to be aggravated by age-related remodelling [4]. Since CT arrhythmias have been documented in patients with and without concomitant CT hypertrophy, it is uncertain if this increase in tissue contributes to the worsening or onset of atrial tachyarrhythmias.

In this case, a patient presenting with palpitations prompted imaging that revealed a pseudo-mass in the right atrium, eventually confirmed as being a hypertrophied CT. The aim of this report is to add to the bank of currently available literature on the topic, in the hopes of streamlining diagnosis in future patients.

## 2. Detailed Case Description

A 58-year-old female patient presented to another medical service for palpitations with a rapid, irregular rhythm that had begun approximately one month earlier, accompanied by elevated blood pressure values. The patient had no cardiovascular risk factors other than the recently diagnosed arterial hypertension and no previously known cardiac pathology.

A 12-lead electrocardiography (ECG) showed a sinus rhythm, heart rate of 60 bpm, no ST-segment modifications and no T-wave modifications. A 24 h Holter ECG showed self-limited episodes of atrial tachycardia, reaching a maximum heart rate of 170 bpm.

**Figure 1:**
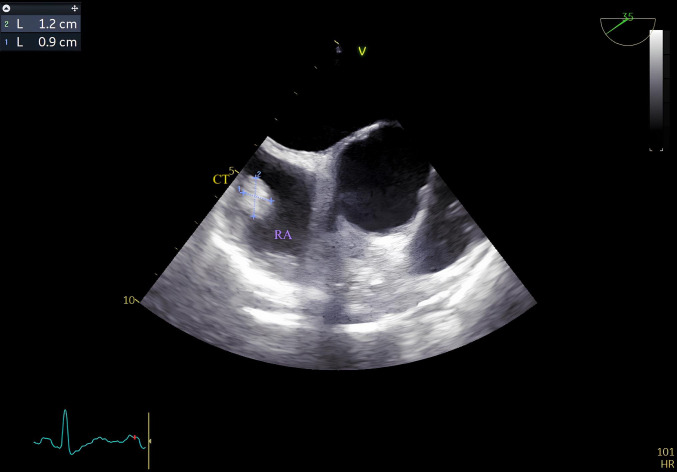
Transesophageal echocardiography (TEE) demonstrating a right atrial mass with dimensions of 12 × 9 mm. CT: crista terminalis, RA: right atrium.

**Figure 2:**
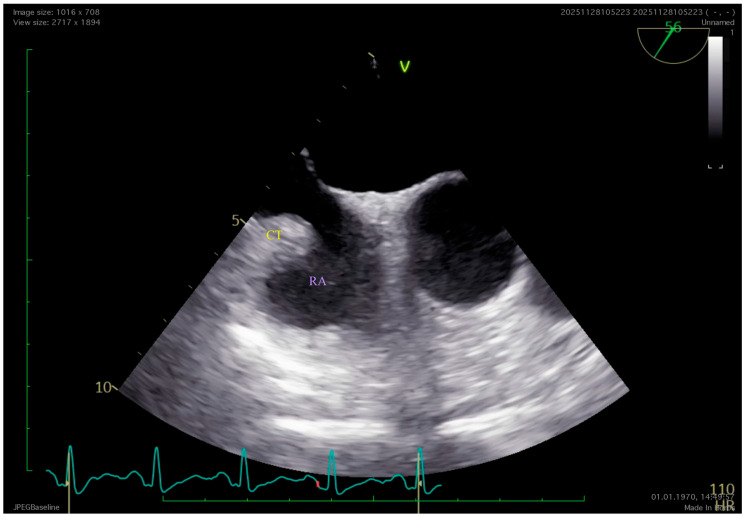
TEE showing a hypertrophied crista terminalis presenting as a right atrial mass. CT: crista terminalis, RA: right atrium.

**Figure 3:**
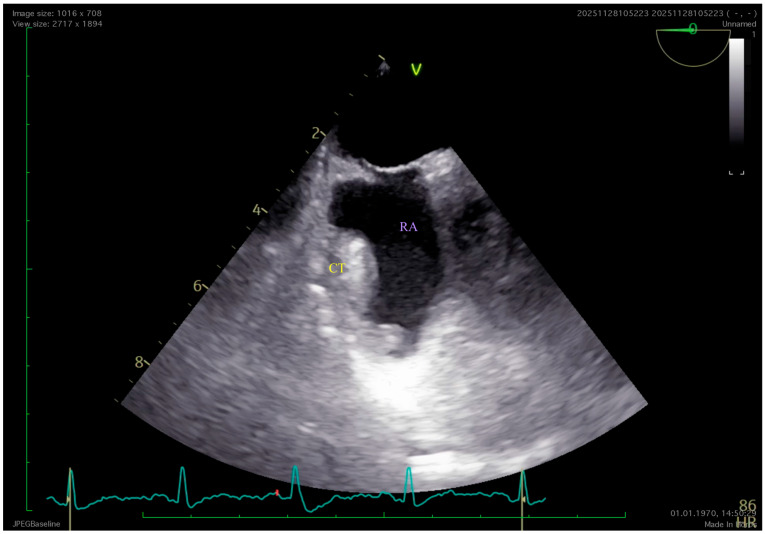
TEE showing a hypertrophied crista terminalis along the lateral right atrial wall; CT: crista terminalis, RA: right atrium.

Initial transthoracic echocardiography (TTE) revealed an echogenic mass within the right atrium, situated along the lateral wall. Left ventricular systolic function (LVEF) was preserved, and right ventricular size and function were within normal limits. There was no evidence of inflow obstruction across the tricuspid valve or significant pericardial effusion. Myocardial kinesis was preserved, without evidence of hypertrophy or structural remodelling. To further characterize the morphology and attachment of the mass, transesophageal echocardiography (TEE) was performed. On TEE, the mass presented as a 12 × 9 mm echogenic and relatively homogeneous structure at the level of the lateral right atrial wall, near the junction of the inferior vena cava (IVC) (Figure 1, Figure 2, Figure 3). The lesion presented a broad base of implantation, with regular margins and no intrinsic mobility. Colour Doppler imaging showed no internal flow, suggesting a non-vascularized structure. While the appearance was non-specific, an initial differential diagnosis of an atrial lipoma was considered, a common benign primary cardiac tumour.

Additional TEE findings included a non-dilated left atrium and a thrombus-free left atrial appendage with preserved Doppler flow. The left ventricle was not dilated, with a preserved ejection fraction (LVEF > 55%) and no regional wall motion abnormalities. The mitral valve leaflets were supple, showing only trace/mild regurgitation. Dimensions of the aortic root and ascending aorta were within normal limits.

**Figure 4:**
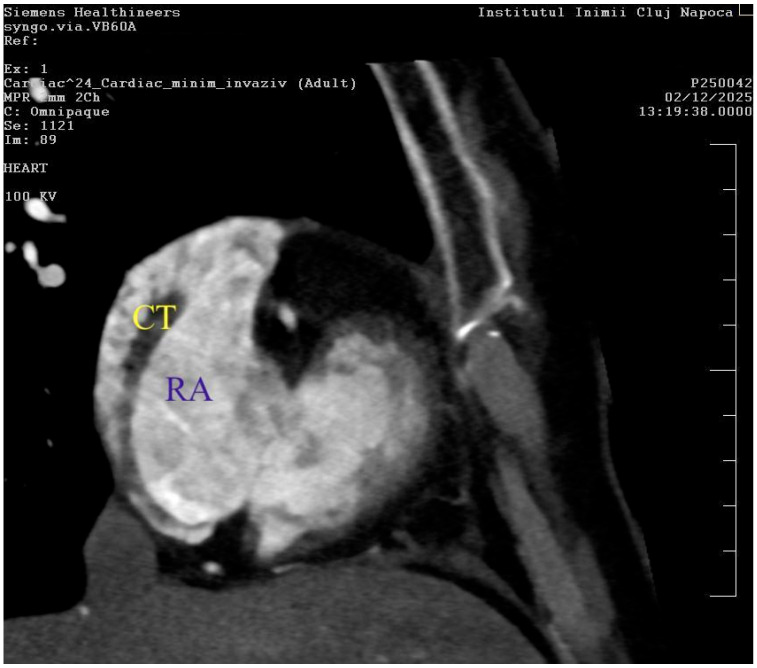
Cardiac computed tomography angiography (CCTA) demonstrates a non-enhancing intracavitary mass within the right atrium, presenting as a well-circumscribed filling defect. CT: crista terminalis, RA: right atrium.

**Figure 5:**
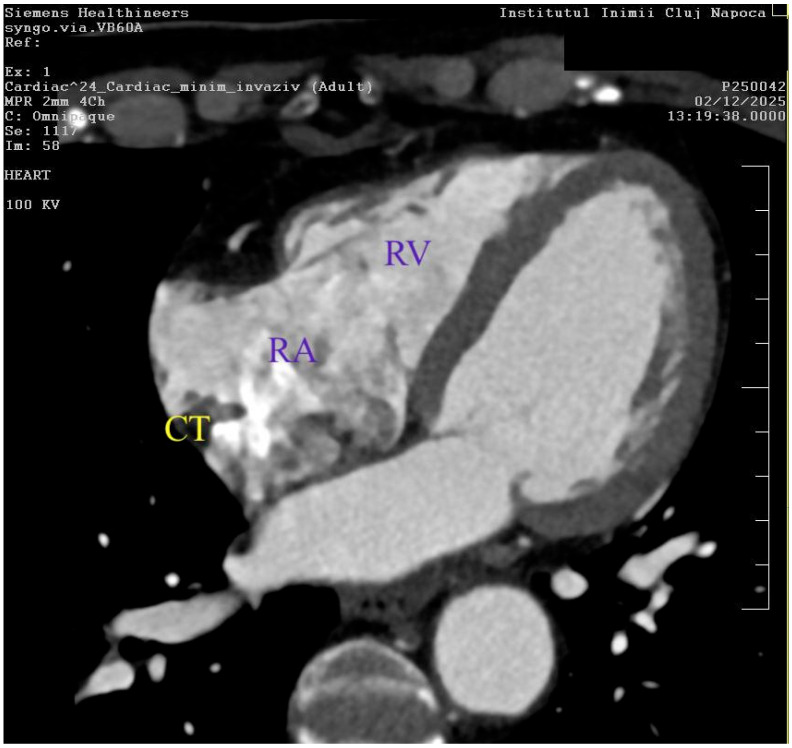
CCTA showing a filling defect within the right atrium, represented by the hypertrophied CT. CT: crista terminalis, RA: right atrium, RV: right ventricle.

Cardiac computed tomography angiography (CCTA) was subsequently performed for better tissue characterization; the results are shown in Figure 4 and Figure 5. The calcium score was 76.3, indicating low cardiovascular risk. Analysis of the coronary arteries revealed right-dominant circulation with mild, non-obstructive atherosclerotic disease. Coronary artery disease (CAD) severity was classified according to the CAD-RADS 2.0 guidelines. The ‘P’ modifier was employed to denote total plaque burden, with P1 indicating mild plaque involvement. Minimal coronary stenosis (1–24%) and a mild plaque burden resulted in a CAD-RADS score of 1/P1. Specifically, the left anterior descending artery (LAD) and the second diagonal branch (D2) exhibited partially calcified and calcified plaques, resulting in minimal luminal stenosis (10–20%). The left main, circumflex, and right coronary arteries were unremarkable. Evaluation of the cardiac morphology revealed non-dilated chambers and no evidence of septal defects.

Notably, the examination confirmed a prominent, hypertrophic CT with a characteristic congenital appearance. Tissue density indicated the mass was not made up of fatty tissue, eliminating the suspicion of a lipoma. There was no evidence of abnormal tissue enhancement or calcification within the right atrium, effectively ruling out a pathological mass. Extracardiac findings were limited to mild paraseptal emphysema in the pulmonary apices and minimal non-calcified atheromatosis of the thoracic aorta.

## 3. Discussion

### 3.1. Crista Terminalis Hypertrophy in the Current Literature

**Figure 6:**
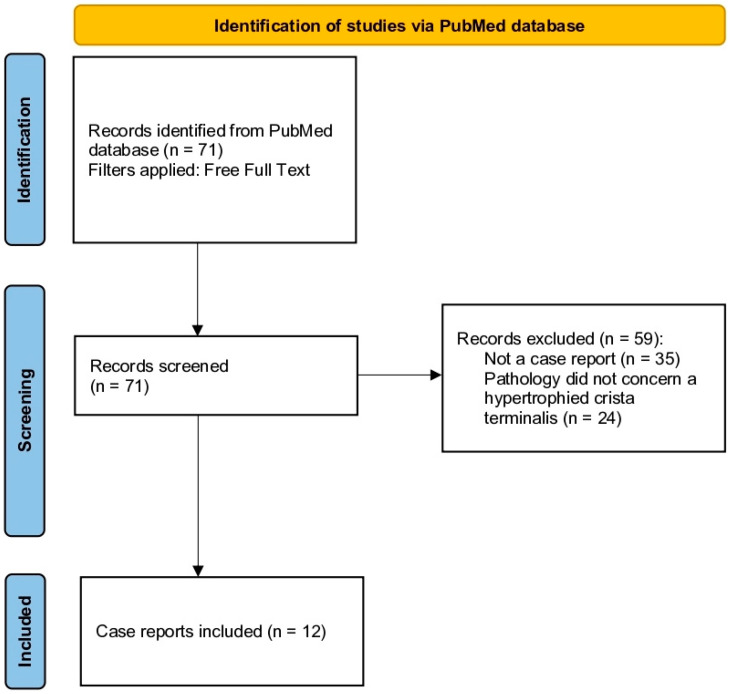
PRISMA flow diagram showing article selection.

A literature review was conducted to identify cases of CT hypertrophy using the PubMed database and keywords “crista terminalis” (Figure 6). An iterative search strategy was employed to ensure maximum capture of relevant cases. To ensure the reproducibility of our literature review and prioritize the most accessible clinical data for the general practitioner, inclusion was limited to free full-text articles. Database results were screened and records that did not present a case report and case reports detailing pathologies other than a hypertrophied CT were excluded. 12 freely available case reports examining this pathology remained [1,2,3,5,6,7,8,9,10,11,12,13].

Table 1 summarizes the sex and age of the patients, whether concomitant arrhythmia was present, and the key imaging techniques used to establish a diagnosis. In the available literature, 66.67% (6/9) of adult patients with crista terminalis hypertrophy were female, 22.22% (2/9) presented a concomitant atrial tachyarrhythmia, and ages ranged from 26 to 79 [1,2,5,6,7,8,9,10,13]. Mean age of diagnosis for the adults included in these case reports was 58.11 ± 17.74 [1,2,5,6,7,8,9,10,13].

Of the nine cases present in adults, only three were recorded in male patients and all three of these patients did not present a concomitant arrhythmia [7,9,13]. This tendency to present silently in males could explain certain outliers among female patients, notably the case report concerning a 32-year-old female with paroxysmal atrial fibrillation [1]. If CT hypertrophy is mainly silent in males, it follows that age at diagnosis is closer to the age at which they would first undergo a full cardiovascular assessment. However, if there does exist a link between a hypertrophied CT and atrial tachyarrhythmias, patients would seek medical advice earlier in life, at the onset of symptoms.

Three of the twelve cases concerned fetuses [3,11,12]. These cases highlight the need to distinguish between the mechanisms by which a prominent CT may arise. The CT could present a failure of complete regression during embryogenesis, or it could present over-development of the structure. Cases in fetuses provide an interesting avenue for following the development of this prominent CT over time. Evong et al. (2018) and Pati et al. (2025) detail follow-up in infancy showing no progression in the size of the CT [3,12]. In the case described by Bhatia et al. (2021), however, the structure showed regression in infancy [11]. Hypertrophied CT is considered to be a benign anatomical variant, therefore close postnatal monitoring is not currently indicated [12]. Nevertheless, knowing a patient presents a congenital right atrial formation can help with the differential diagnosis of an intracardiac mass upon reaching adulthood.

#### 3.1.1. Association with Atrial Tachyarrhythmia

The CT is a well-documented arrhythmogenic substrate, implicated in a broad spectrum of atrial tachyarrhythmias. In the literature, its unique electrophysiological properties have been implicated in the pathogenesis of focal atrial tachycardia, atrial flutter, and atrial fibrillation [4,14,15]. The CT accounts for two-thirds of right atrial arrhythmias in patients without structural cardiac abnormalities [1]. While the CT is involved in various arrhythmias, our patient’s presentation specifically involved self-limiting episodes of focal atrial tachycardia, consistent with the enhanced automaticity, triggered activity, and micro-reentry often found in this area [16].

Clinical presentation of atrial tachyarrhythmias can, such as was the case here, lead to cardiac imagery that prompts the discovery of a crista terminalis hypertrophy. Epidemiologically, the literature shows that patients are more commonly over 50 and female, with Morris et al. (2019) placing mean age at presentation at 56.9 ± 1.5 [4]. Our case report adds to this available data, as our patient was also over 50 and female, and presented concomitant atrial tachycardia. The CT being a structure that arises during embryonic development, its discovery in young patients is not rare. Salim et al. (2016) presented the case of a 32-year-old woman with paroxysmal atrial fibrillation, for whom a prominent crista terminalis was discovered during her pre-operative cardiology assessment [1]. Pati et al. (2025), Bhatia et al. (2021), and Evong et al. (2018) detail the discovery of right atrial masses in fetuses that were later confirmed to be prominent crista terminalis [3,11,12]. In the fetal and neonatal context, the differential diagnosis of right atrial masses is critical, as other pathologies such as cardiac rhabdomyoma can present with supraventricular extrasystoles, requiring surgical resection for symptom resolution [17]. The case of other right atrial masses leading to symptoms also favours the notion that CT hypertrophy, when it reaches a certain thickness or a precise anatomical region, may cause arrhythmias. In older patients, the association between the CT and atrial tachyarrhythmia is thought to be linked to age-related remodelling leading to slowed conduction [4]. This indicates that a prominent crista terminalis may be a risk factor for developing arrhythmias later in life.

The CT is an arrhythmogenic substrate for both micro- and macro-reentrant arrhythmias [18]. Physio-pathological mechanisms behind the arrhythmogenic potential of the CT include its relation to the sino-atrial (SA) node, conduction anisotropy, and cellular automaticity [4,19,20].

The CT corresponds internally to the sulcus terminalis, a structure that houses the SA node [20]. Conduction velocity in the CT is high when compared to other regions of the right atrium and the SA node [21]. Abnormal thickening of the CT may therefore alter the way electrical impulses spread from the SA node. When measuring atrial wall thickness, the CT is found to be significantly thicker than the surrounding tissue [14]. Furthermore, the most common origin of atrial tachycardia is located at the mid-third of the structure, with superior and mid-sites being significantly more frequent origins than the inferior third [4]. Critically, these areas, the superior and mid-third of the CT, overlap with the main SA node exits and earliest atrial activation sites [18]. In fact, the study of ex vivo human hearts showed that most superior, mid-lateral, and inferior SA node exits were often located along the CT [18]. Mapping by Zhao et al. (2023) has identified that right atrial drivers of atrial fibrillation are consistently anchored on the CT at both superior and inferior sections of the right atrium [14].

Conduction anisotropy across the CT means that conduction velocity is fast longitudinally but slow transversely [19]. Sánchez-Quintana et al. (2002) describe how the CT acts as a natural barrier to transverse conduction, favorizing the appearance of macro-reentrant circuits, as seen in atrial flutter [19]. A more recent study by Morris et al. (2019) suggests that this same anisotropy leads to micro re-entrant circuits [4]. Slow transverse conduction due to poor transverse cell-to-cell coupling thus leads to both micro- and macro-reentrant circuits [18]. A fundamental type of macro-reentrant atrial tachyarrhythmia is atrial flutter [15]. Typical atrial flutter arises from a circuit that is limited by the tricuspid annulus anteriorly, the CT and eustachian ridge posteriorly, and the cavotricuspid isthmus (CTI) inferiorly [15]. Thus, atrial flutter is further categorized as clockwise or anticlockwise, according to the direction of flow, and CTI-dependent or non-CTI-dependent [15]. Bhargav et al. (2025) describe a case of counterclockwise macro-reentry around the IVC with breakthrough at the lower end of the CT causing atypical atrial flutter [22]. A possible complication of lateral tunnel Fontan surgery is a macro-reentrant circuit around the baffle-CT suture line [23]. Age-related remodelling further contributes to this anisotropy due to further loss of gap junction proteins, already poorly expressed in the CT [4]. Thus, modification of the tissue of the CT can disrupt local electrical transmission.

Cellular automaticity in the CT is due to greater hyperpolarization-activated cyclic nucleotide-gated (HCN) channel expression than in the surrounding myocardium [4]. While macro-reentrant tachycardia is often associated with structural heart disease, heart failure or ischemic cardiomyopathy, focal atrial tachycardia frequently occurs in healthy individuals without structural abnormalities [24]. Focal atrial tachycardias arise at the CT in over two-thirds of cases [16]. The three main mechanisms driving focal atrial tachycardia within this region are: enhanced automaticity, triggered activity, and micro-reentry [16]. When HCN channels are overactive, or if surrounding tissue is remodelled, focal automaticity can be triggered, leading to atrial tachycardias [4]. Specifically, enhanced automaticity results from an accelerated spontaneous phase 4 depolarization upslope [16]. When the cell is hyperpolarized, the activation of HCN channels triggers an influx of sodium and potassium that allows the cell to reach threshold [25]. Alternatively, triggered activity involves calcium channel dysfunction and is the result of post-depolarization focal electrical events that produce repeat depolarization in phases 2, 3 or 4 [16,24]. Meanwhile, micro-reentry occurs between adjacent substrates with varying conduction velocities and repolarization rates, such as fibrotic areas or prior ablation scars, much like is the case for conduction anisotropy across the CT [16].

Catheter ablation was shown to be successful in treating these tachyarrhythmias [4,18]. With the addition of 3D mapping, the ablation success rate for atrial tachycardia is as high as 98.5% [4]. However, in the case of atrial fibrillation, a retrospective study by Pan et al. (2023) showed that CT thickness is an independent predictor of recurrence after radiofrequency ablation [26]. Specifically, a median CT thickness of 3.70 mm (IQR 3.30, 4.40) was associated with atrial fibrillation recurrence, compared to 3.40 mm (IQR 3.10, 3.90) in non-recurrent cases [26]. A subsequent study by Pan et al. (2024) stratified atrial fibrillation patients undergoing radiofrequency ablation into paroxysmal and persistent cohorts [27]. While a significant association between CT thickness and arrhythmia recurrence was observed across both groups, multivariate logistic regression analysis identified CT thickness as an independent predictor of recurrence specifically within the persistent atrial fibrillation population [27]. Although these studies focused on atrial fibrillation, the CT is frequently highlighted in the pathogenesis of various right atrial tachyarrhythmias. Furthermore, ultra-high-resolution 3D mapping has recently demonstrated the complexity of this barrier, revealing that unusual posterior transverse conduction can persist across the CT even after standard ablation procedures [28]. Possible risks of catheter ablation include proximity of the phrenic nerve to the right atrium [29]. However, this risk can be reduced by performing the ablation during inspiratory hold and using concomitant phrenic nerve monitoring [29].

While the anatomical prominence of the crista terminalis was clearly visualized on CCTA and TEE, its definitive role as the arrhythmogenic substrate in this patient remains speculative. In the absence of invasive electrophysiological mapping, we cannot definitively confirm that the patient’s episodes of self-limiting atrial tachycardia originated specifically from this fibromuscular ridge. However, the correlation between the mass-like appearance of the CT on imaging and the patient’s clinical presentation of atrial tachycardia is consistent with the described role of the CT as a frequent origin of focal atrial tachycardia [4].

#### 3.1.2. Diagnostic Imaging and Differential Diagnosis

The diagnostic workup of a suspected cardiac mass must follow a systematic, stepwise escalation from first-line screening to advanced tissue characterization. On TTE, the CT is not always clearly visible [3]. When hypertrophied, it can mimic pathological right atrial masses, such as thrombi or tumours [3]. Other important right atrial mass mimics include the Eustachian valve, cor triatriatum dexter (CTD), Thebesian valve, atrial septal aneurysms, and the Chiari network [1].

##### First-Line Assessment and Risk Stratification

Following the detection of a suspicious right atrial mass on TTE, the primary clinical objective is to exclude malignancy. Diagnostic accuracy can be enhanced by utilizing standardized echocardiographic markers, such as those integrated into the Diagnostic Echocardiographic Mass (DEM) score [30,31]. This scoring system stratifies malignancy risk by evaluating six key criteria: myocardial infiltration, polylobate morphology, moderate-to-severe pericardial effusion, tissue heterogeneity, sessile attachment, and non-left heart localization [30,31]. Notably, Paolisso et al. (2023) demonstrated that a DEM score ≥ 3 yielded a diagnostic accuracy of approximately 90% for malignant cardiac masses [31].

##### Second-Level Imaging

Compared to TTE, TEE offers enhanced spatial resolution and superior visualization of cardiac structures. Real-time 3D TEE is particularly valued for its precision in quantifying right atrial anatomy [32]. Hassan et al. (2023) demonstrated that annular dimensions measured using 4D TEE correlate strongly with multi-detector computer tomography (MDCT) findings, establishing it as a reliable alternative for structural assessment [33].

Clearly defining the echocardiographic characteristics of a hypertrophied CT reduces diagnostic uncertainty and may preclude the need for additional imaging in straightforward cases. A hypertrophied CT typically appears as a C-shaped muscular band or a well-defined fibromuscular ridge with a broad base of insertion, located on the posterolateral wall of the right atrium [2]. The CT originates near the medial border of the superior vena cava (SVC) and extends along the posterolateral wall towards the IVC [34]. It is typically isoechoic to the adjacent myocardium [6]. The CT serves as the boundary between the smooth venous component and the trabeculated regions containing the pectinate muscles of the right atrial appendage (RAA) [35,36]. While formal diagnostic criteria for what constitutes a prominent CT are currently lacking, a normal adult CT has a mean length of 51 ± 9 mm and a thickness of 5.5 mm [34]. In the present case, the identified mass measured 12 × 9 mm, exceeding the average thickness and thus mimicking the morphology of a sessile intra-atrial tumour. The CT does not enhance with contrast, and tissue Doppler imaging shows no aberrant flux [34]. The structure exhibits phasic changes in shape during atrial systole but lacks independent mobility, moving in synchronicity with the cardiac cycle [6].

Using TEE, the CT can be differentiated from other anatomical variants. The Chiari network, Eustachian valve, and CTD result from incomplete regression of the right sinus venosus valve [37]. The Chiari network presents as a mobile, fenestrated, and thread-like structure [38]. Phuyal et al. (2025) recently highlighted how its high mobility and web-like appearance often lead to its initial misidentification as a thrombus or infective vegetation, mirroring the diagnostic challenges posed by a prominent CT [38]. A prominent Eustachian valve is characterized as a crescent-shaped fold of endocardium that extends from the anterior border of the inferior vena cava (IVC) [37]. This structure is often found in patients with a patent foramen ovale, in which case it can facilitate right-to-left shunting and increase the risk of paradoxical embolism [39]. Complete CTD manifests as a membrane that divides the right atrium into two compartments and severely obstructs blood flow, whereas incomplete CTD is only a partial membrane, generating no or mild obstruction [37]. CTD can be associated with atrial septal defects, contributing to a left-to-right shunt [37]. The Thebesian valve, an anatomical variant located at the coronary sinus opening, may interfere with coronary sinus cannulation, complicating certain cardiovascular procedures [40]. The morphology of the Thebesian valve is highly variable, with six main types having been described in the literature, the most frequent of which is semilunar [40]. Atrial septal aneurysms are mobile saccular dilations of the atrial septum that have been tentatively linked to atrial arrhythmias, cryptogenic stroke, and embolic events [41].

##### Tertiary Modalities and Further Risk Stratification

Further imaging using CCTA or Cardiac magnetic resonance imagery (CMR) provides definitive tissue characterization [7]. CCTA offers high special resolution and rapid image acquisition [7]. It is particularly useful for identifying the relationship between the CT and the SVC, as well as its role in conduction blocks [7,42]. While it involves ionizing radiation and iodinated contrast, it is often more accessible and faster than CMR [7].

CMR provides excellent contrast resolution and does not expose the patient to ionizing radiation [7,43]. It differentiates the CT from neoplasms by confirming signal intensity identical to normal myocardium and demonstrating an absence of abnormal gadolinium enhancement [9]. However, its use is limited by higher costs, longer scan times, and incompatibility with certain intracardiac devices [7].

In this case, while TEE raised suspicion for a lipoma due to its echogenicity and sessile attachment, subsequent CCTA provided definitive characterization. The mass was identified as a prominent, hypertrophic CT, a well-documented pseudotumour of the right atrium. The definitive diagnosis was supported by its characteristic anatomical course and lack of abnormal contrast enhancement or fatty infiltration.

The CMR Mass Score further assists clinicians in evaluating malignancy risk [44]. This score weighs the following parameters: infiltration, polylobate morphology, pericardial effusion, sessile attachment, contrast perfusion, and heterogeneity enhancement [44]. A cutoff score of ≥5 provides 92% sensibility and 96% specificity in identifying malignant cardiac masses [44].

Finally, in complex cases, 18-FDG PET/CT may be utilized. A hypertrophied CT typically shows no abnormal FDG uptake, helping rule out malignant tumours or inflammatory masses [6,9]. However, some cases have noted focal uptake that can masquerade as a tumour thrombus [8].

#### 3.1.3. Proposed Diagnostic Algorithm for Prominent Crista Terminalis

**Figure 7:**
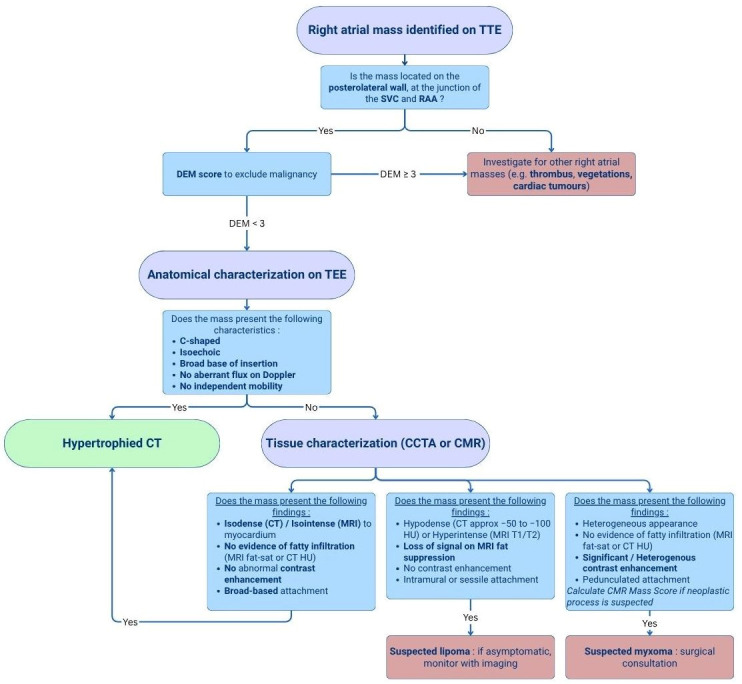
Diagnostic algorithm for prominent crista terminalis.

We propose a standardized diagnostic algorithm to assist clinicians in the efficient identification of a prominent CT and to streamline the differential diagnosis of right atria masses (Figure 7). Early characterization using TEE may preclude the need for more invasive or costly investigations, optimizing both clinical timelines and resource allocation.

Upon the initial detection of a right atrial mass using TTE, the primary diagnostic step is precise anatomical localization. A mass located on the posterolateral wall, at the junction between the SVC and the RAA, is highly suggestive of a hypertrophied CT. Conversely, masses in atypical locations should prompt immediate investigation into alternative aetiologies, including thrombi or vegetations.

TEE provides superior resolution for anatomical characterization. The presence of a C-shaped, isoechoic mass with a broad base of insertion, no independent mobility, and no aberrant flux on Doppler imaging orients the diagnosis towards a prominent CT. Should diagnostic ambiguity persist, CMR and CCTA are the established gold standards for definitive tissue characterization. As delineated in Figure 7, the algorithm facilitates the differentiation between CT hypertrophy, cardiac lipoma, and myxoma based on their typical features on CCTA or CMR. We specifically recommend the integration of the DEM score and CMR Mass Score within this pathway (Figure 7) to ensure that suspicious features are quantified rather than qualitatively assessed, thereby reducing the risk of misdiagnosing a malignant process as a benign variant.

### 3.2. Significant Contributions

Our case significantly contributes to the sparse literature on hypertrophic CT by reinforcing a specific clinical and demographic phenotype. The patient’s profile, a 58-year-old female, aligns precisely with the demographic data shown in our literature review. The mean age of diagnosis at 58 and the female predominance noted in the aforementioned review support that a prominent CT should be considered a primary differential in women in their sixth decade presenting with unexplained palpitations and a right atrial finding.

Furthermore, while existing literature focuses on either the importance of imaging or the electrophysiology of the CT, our report bridges these two domains. We demonstrate the diagnostic dilemma, how a benign anatomical structure can lead to the suspicion of a high-risk mass on initial imagery, and highlight the possibility that the hypertrophy of this structure could lead to electrical instability. By synthesizing recent data, such as the multivariate analysis by Pan et al. (2024) [27], which identifies CT thickness as an independent predictor of recurrence in persistent atrial fibrillation, we move beyond the decade-old observations summarized in recent reviews [45].

This study contributes to the clinical framework for evaluating right atrial masses by advocating for a structured multimodality imaging approach. We underscore the necessity of escalating from TTE screening to TEE for high-resolution morphological assessment, while establishing the utility of 3D and 4D TEE in providing the volumetric clarity required to confirm anatomical variants. Furthermore, our work validates CCTA as a confirmatory tool that offers the dual advantage of high-resolution tissue characterization and simultaneous coronary assessment. As such, it allows clinicians to definitively exclude malignancy or benign pseudotumors, such as lipomas, through the evaluation of contrast enhancement and fatty infiltration. This workflow is complemented by CMR, the gold standard for tissue characterization, and 18F-FDG PET/CT, which provides metabolic data to differentiate between malignant, inflammatory, or metabolically inactive masses like a hypertrophied CT.

### 3.3. Limitations

A notable limitation of this case report is the lack of invasive electrophysiological data. As the patient’s episodes were self-limiting and successfully managed with pharmacological therapy, catheter ablation and high-resolution 3D mapping were not clinically indicated. Consequently, the proposed bridge between imaging findings and the electrophysiological mechanism remains a theoretical correlation rather than a confirmed causality.

A significant limitation of this literature review is the restriction to free, full-text articles. This approach may have excluded pertinent, high-impact case reports and longitudinal studies available only through subscription-based journals. Consequently, the synthesis of current evidence provided here should be interpreted as a representative snapshot rather than an exhaustive systematic analysis. Future reviews would benefit from a broader search strategy to ensure the inclusion of all the latest significant data.

## 4. Conclusions

The CT is a benign, physiological structure located in the right atrium. However, hypertrophy and the arrhythmogenic potential of this structure can lead to clinical repercussions.

A hypertrophied CT mimics a right atrial mass, requiring a mandatory differential diagnosis with life-threatening aetiologies such as thrombi and tumours [3]. TEE is a powerful tool that provides superior tissue visualization to TTE; however, clear diagnostic criteria for identifying hypertrophied CT need to be established. We propose a diagnostic algorithm that utilizes a stepwise approach to guide clinicians through the imaging pathway. Currently, advanced cross-sectional imaging by CMR or CCTA is valuable, allowing for greater tissue characterization and confirming the diagnosis of CT hypertrophy [7]. In the future, hypertrophic CT identification will likely necessitate only 3D or 4D TEE, further accelerating the time it takes to establish a diagnosis.

The CT is frequently the origin of atrial tachyarrhythmias, through mechanisms such as anisotropy and cellular automaticity, as well as through its anatomical proximity to the SA node [4,19,20]. Steps have been taken towards better understanding the link between CT thickness and arrhythmogenic potential, showing that the recurrence of atrial fibrillation post-ablation is more frequent once CT thickness surpasses 3.70 mm [26]. Further research is needed to properly understand the relationship between CT hypertrophy and the induction of arrhythmogenic events. While pharmacological success precluded the need for invasive electrophysiological studies in our patient’s case, such studies remain the gold standard for confirming the relationship between anatomical landmarks like the crista terminalis and specific tachyarrhythmia circuits. Future reports combining advanced imaging with 3D mapping will be essential to further define this bridge between imaging and electrophysiology.

In conclusion, our case highlights that a hypertrophied CT should be considered in the differential diagnosis of a right atrial mass, especially in female patients in the sixth decade with a history of atrial tachyarrhythmia. Use of multi-modal imaging to confirm this benign anatomical variant can spare the patient from unnecessary and potentially invasive interventions.

## Figures and Tables

**Table 1:** Epidemiological data and presence of concurrent arrhythmia in published case reports concerning crista terminalis hypertrophy.

References	Sex (F/M)	Age	Concomitant Arrhythmia	Key Imaging
Pati et al. (2025) [12]	NR ^1^	Fetus (32 w)	NR	Prenatal ultrasound
Nalawade et al. (2024) [6]	F	65	NR	Real-time 3D TEE ^2^
Shoji et al. (2024) [13]	M	57	NR	Real-time 3D TEE
Ghesani et al. (2023) [8]	F	79	NR	18F-FDG PET-CT ^3^, TEE
Bhatia et al. (2021) [11]	NR	Fetus (23 w)	NR	Prenatal ultrasound
Lakhani et al. (2021) [7]	M	78	NR	CMR ^4^
Ahmed et al. (2020) [5]	F	59	Atrial fibrillation	CCTA ^5^
Evong et al. (2018) [3]	NR	Fetus (20 w)	NR	Prenatal ultrasound
Wang et al. (2018) [9]	M	54	NR	Cardiac PET/MRI ^6^
Salim et al. (2016) [1]	F	32	Paroxysmal atrial fibrillation	CMR
Na et al. (2011) [10]	F	73	NR	TEE
Salustri et al. (2010) [2]	F	26	NR	TEE

^1^ NR = not reported; ^2^ Transesophageal echocardiography; ^3^ 18F-fluorodeoxyglucose positron emission tomography; ^4^ Cardiac magnetic resonance imagery; ^5^ Cardiac computed tomography angiography; ^6^ Positron Emission Tomography/Magnetic resonance imagery.

## Data Availability

The original contributions presented in this study are included in the article. Further inquiries can be directed to the corresponding author.

## Abbreviations

The following abbreviations are used in this manuscript:

CT	Crista terminalis
TTE	Transthoracic echocardiography
TEE	Transesophageal echocardiography
CCTA	Cardiac computed tomography angiography
CMR	Cardiac magnetic resonance imagery
ECG	Electrocardiography
LVEF	Left ventricular systolic function
SA	Sino-atrial
CAD	Coronary artery disease
CAD-RADS	Coronary Artery Disease Reporting and Data System
LAD	Left anterior descending artery
CTI	Cavotricuspid isthmus
HCN	Hyperpolarization-activated Cyclic Nucleotide-gated
CTD	Cor triatriatum dexter
SVC	Superior vena cava
IVC	Inferior vena cava
DEM	Diagnostic echocardiographic mass
MDCT	Multi-detector computer tomography
RAA	Right atrial appendage
18F-FDG PET-CT	Fluorine-18 Fluorodeoxyglucose Positron Emission Tomography/Computed Tomography
